# Seasonal Variation and Severity of Acute Abdomen in Japan: A Nine-Year Retrospective Analysis

**DOI:** 10.3390/jpm11121346

**Published:** 2021-12-10

**Authors:** Hidero Yoshimoto, Kazuma Yamakawa, Yutaka Umemura, Kensuke Fujii, Eriko Nakamura, Kohei Taniguchi, Keitaro Tanaka, Akira Takasu, Kazuhisa Uchiyama

**Affiliations:** 1 Department of Surgery, Osaka Medical and Pharmaceutical University, Takatsuki 569-8686, Japan; hidero.yoshimoto@ompu.ac.jp (H.Y.); kensuke.fujii@ompu.ac.jp (K.F.); kohei.taniguchi@ompu.ac.jp (K.T.); keitaro.tanaka@ompu.ac.jp (K.T.); uchi@ompu.ac.jp (K.U.); 2 Department of Emergency Medicine, Osaka Medical and Pharmaceutical University, Takatsuki 569-8686, Japan; eriko.nakamura@ompu.ac.jp (E.N.); akira.takasu@ompu.ac.jp (A.T.); 3 Osaka General Medical Center, Division of Trauma and Surgical Critical Care, Osaka 558-8558, Japan; plum00022@gmail.com; 4 Department of Surgery, Kasaoka Daiichi Hospital, Kasaoka 714-0043, Japan; 5 Translational Research Program, Osaka Medical and Pharmaceutical University, Osaka 569-8686, Japan

**Keywords:** nationwide database study, sepsis, organ dysfunction scores

## Abstract

The seasonal incidence of acute abdomens, such as appendicitis, is reportedly more common in summer but is reported less frequently in Asia. Additionally, seasonal variations in the severity of acute abdomens have been evaluated insufficiently. This study evaluated the seasonal variations in the incidence and severity of acute abdomens in Japan. This retrospective observational study used a multicenter database containing data from 42 acute hospitals in Japan. We included all patients diagnosed with acute appendicitis, diverticulitis, cholecystitis, and cholangitis between January 2011 and December 2019. Baseline patient data included admission date, sequential organ failure assessment score, presence of sepsis, and disseminated intravascular coagulation. We enrolled 24,708 patients with acute abdomen. Seasonal admissions for all four acute abdominal diseases were the highest in summer [acute appendicitis, (OR = 1.35; 95% CI = 1.28–1.43); diverticulitis, (OR = 1.23; 95% CI = 1.16–1.31; cholecystitis (OR = 1.23; 95% CI = 1.11–1.36); and cholangitis (OR = 1.23; 95% CI = 1.12–1.36)]. The proportion of patients with sepsis and disseminated intravascular coagulation as well as the total SOFA score for each disease, did not differ significantly across seasons. Seasonal variations in disease severity were not observed.

## 1. Introduction

Acute abdomen is one of the most common emergency conditions encountered in daily clinical practice. The severe acute abdomen can progress to generalized peritonitis, sepsis, and disseminated intravascular coagulation (DIC) [1,2]. In previous studies, the incidence of acute appendicitis, diverticulitis, acute cholecystitis, and acute cholangitis were reported to be 76.2–93.8/100,000, 47.4/100,000, 88.1–108.5/100,000, and 6.4–7.2/100,000, respectively [3,4,5], while the corresponding mortality rates were 0.12–5.4%, 0.24–0.33%, 0.7%, and 2.4–8.4%, respectively [6,7,8,9].

Seasonal variations have been reported in the incidence of several diseases. For example, cardiac arrest and asthma have been reported to be most common in winter [10,11]. Similarly, several studies have reported that the frequency of acute appendicitis was higher in summer [12,13], and some have reported that admissions for diverticulitis and acute cholecystitis were more common in summer [12,14,15]. However, seasonal variations in the severity of acute abdomen have been reported less frequently, especially in Japan. Since the four seasons in Japan are clearly separated compared to the other countries where previous studies were conducted, the analysis of seasonal variations in Japan may yield clear distinctions among seasons. For example, the average temperature in Tokyo is 14.7 degrees Celsius (11.1–19.7) degrees during spring, 26.4 (22.9–27.4) degrees Celsius during summer, 19.1 (14.1–22.9) degrees Celsius during fall, and 6.3 (5.7–7.3) degrees Celsius during winter. Therefore, we attempted to analyze the seasonal trends in acute abdomen using a nationwide database used for claiming medical fees in Japan. Specifically, we aimed to investigate the monthly and seasonal variations in the frequency and severity of acute abdominal conditions, namely, acute appendicitis, diverticulitis, acute cholecystitis, and acute cholangitis.

## 2. Materials and Methods

### 2.1. Design and Setting

This retrospective observational study was conducted using routinely collected data from electronic medical records provided by Medical Data Vision (MDV, Tokyo, Japan). The MDV database contains electronic health insurance claims and diagnosis procedure combination (DPC) payment system data from approximately 400 acute hospitals, accounting for approximately 23% of the total claims in Japan and including approximately 30 million patients until October 2019. The database includes data on age, sex, laboratory values, admission date, primary diagnoses, concomitant diagnoses, complication diagnoses, medical procedures, prescriptions, drug administration, discharge status, and hospital length of stay. This study included patient data from 42 acute hospitals (approximately 1.2% of all the acute hospitals in Japan) with laboratory data among all acute hospitals registered in the MDV database. Diagnoses were recorded based on the International Classification of Diseases Tenth Revision (ICD-10) codes.

This study was conducted in accordance with the principles of the Declaration of Helsinki. The study protocol was approved by the Institutional Review Board of Osaka General Medical Center, Osaka, Japan (approval no. #S201916015). Informed consent was not required because of the anonymous nature of the retrospective data.

### 2.2. Study Population

**Figure 1:**
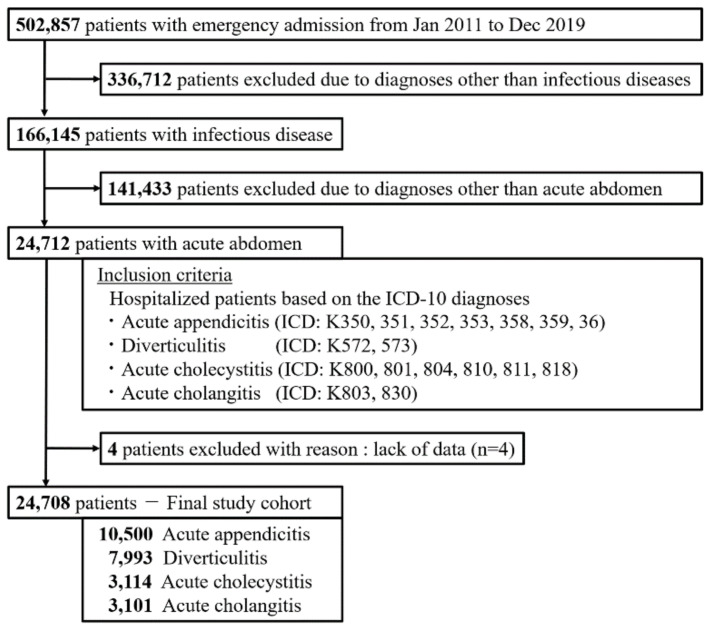
Patient flow. Flowchart of patients admitted to this study from 2011 to 2019.

The flowchart outlining patient selection for this study is shown in Figure 1. We identified all adult patients who required unplanned hospital admission and were diagnosed with an infection between 1 January 2011 and 31 December 2019. In this study, infection was defined by the inclusion of any of the ICD-10 infection codes previously proposed by the Institute for Health Metrics and Evaluation (IHME) [16] in the primary diagnosis or the diagnosis that triggered hospitalization. Among these patients, those diagnosed with acute appendicitis (ICD-10 codes K350, K351, K352, K353, K358, K359, and K36), diverticulitis (K572 and K573), acute cholecystitis (K800, K801, K804, K810, K811, and K818), or acute cholangitis (K803 and K830), regardless of emergency surgery during hospitalization, were included in the study. Patients with missing age data were excluded from the analysis.

### 2.3. Data Collection

We collected the following data for evaluation of baseline patient characteristics: Age, sex, date of admission, Charlson comorbidity index (CCI), [17] Sequential Organ Failure Assessment (SOFA) score and SOFA sub-scores, intensive care unit admission, use of catecholamine, surgery with general anesthesia, and underlying Sepsis-3 and DIC. Sepsis-3 was defined by an increase of 2 or more points from the total SOFA score on admission, which was calculated retrospectively. In this study, we used the modified SOFA score listed in Table S1, which omits cardiovascular subscore 1 (mean arterial pressure < 70 mmHg) and respiratory subscore 4 (PaO_2_/F_I_O_2_ < 100), because data for these variables were not provided in the MDV database. The Japan coma scale, which is used for calculating neurological sub-scores instead of the Glasgow coma scale, has four main grades (grade 0 = alert; grade 1 = possible verbal response without any stimulation, not lucid; grade 2 = possible eye-opening, verbal, and motor response upon stimulation; and grade 3 = no eye-opening and coma upon stimulation). DIC diagnoses were based on ICD-10 codes (D65, O450, O460, O723, and O081) instead of the established diagnostic criteria for DIC. We collected the following data on general outcomes: in-hospital mortality, length of hospital stay, and emergency surgery with general anesthesia. To examine seasonal variations, we defined the seasons as follows: spring (1 March–31 May), summer (1 June–31 August), fall (1 September–30 November), and winter (1 December–28 February).

### 2.4. Statistical Analysis

To compare seasonal variations in the frequency and severity of acute abdomen (acute appendicitis, diverticulitis, acute cholecystitis, and acute cholangitis), analyses were performed using a nonparametric test, an extension of the Wilcoxon rank test for continuous variables, and a logistic regression test. Categorical variables are presented as numbers and percentages, while continuous variables were presented as median and interquartile ranges. All statistical inferences were performed using a 2-sided *p* value at the 5% significance level. All analyses were performed using JMP 15.0 software (SAS Institute, Tokyo, Japan).

## 3. Results

### 3.1. Study Population

The total number of infectious disease inpatients during the study period was 166,145. After applying the inclusion and exclusion criteria, 24,708 patients with acute abdomen were included in this study. The diagnoses on admission were as follows: acute appendicitis (42.5%, *n* = 10,500), diverticulitis (32.3%, *n* = 7993), acute cholecystitis (12.6%, *n* = 3114), and acute cholangitis (12.6%, *n* = 3101). The baseline patient characteristics for each diagnosis are shown in Table 1.

The incidence of acute appendicitis was higher in younger people; however, diverticulitis, acute cholecystitis, and acute cholangitis were more common in older adults. Sepsis-3 as an underlying condition was more frequent in acute cholecystitis and acute cholangitis. The outcome measures for all patients for each diagnosis are presented in Table 2. The mortality rates for all four diseases were higher in older adults. In particular, acute cholecystitis and acute cholangitis showed higher mortality rates than the other conditions.

### 3.2. Monthly and Seasonal Variations in Admissions

**Figure 2:**
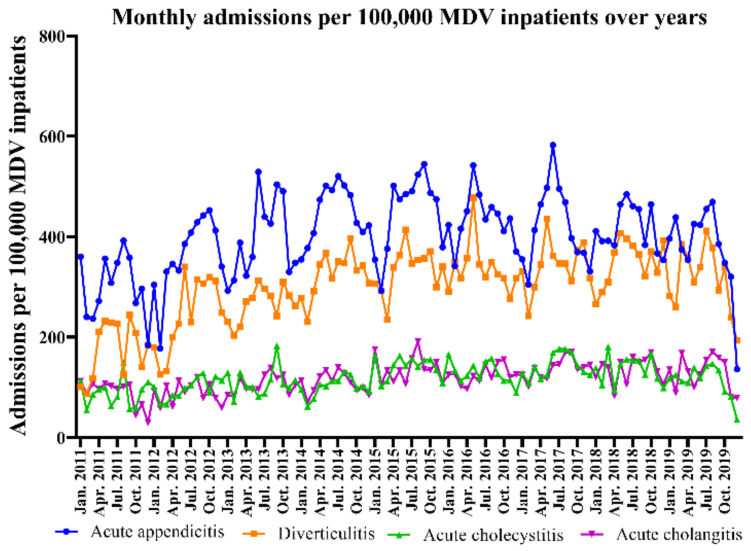
Monthly admissions per 100,000 MDV inpatients over the years. MDV, medical data vision.

The monthly admission rate per 100,000 MDV inpatients for each disease is shown in Figure 2. From 2011 through 2019, the rate of acute abdomen admissions was the lowest in February (6660.8/100,000 MDV inpatients) and the highest in August (9573.8/100,000 MDV inpatients), showing a 43.7% increase. Similarly, the monthly admission rates of individual acute abdomen conditions were the lowest in December to February and the highest in May to June.

**Figure 3:**
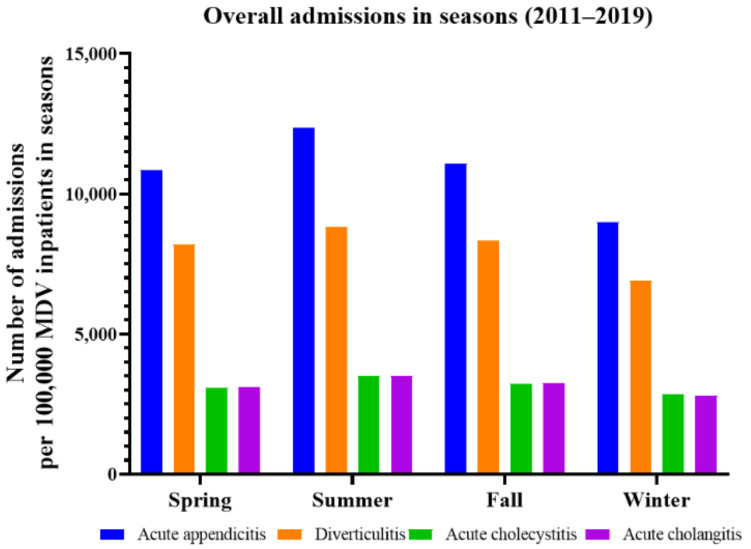
The seasonal admissions for acute appendicitis, diverticulitis, acute cholecystitis, and acute cholangitis were clearly higher in summer than winter, with meaningful differences. MDV, medical data vision.

The seasonal admission rates per 100,000 inpatients with MDV for each disease are shown in Figure 3. The rate of admissions for acute abdomen was the lowest in winter (21,581.9/100,000 MDV inpatients) and the highest in summer (28,230.3/100,000 MDV inpatients), representing a 30.8% increase.

The analysis of seasonal admissions for each diagnosis is listed in Table 3. The rate of seasonal admissions for acute appendicitis was the highest in summer (odds ratio (OR) = 1.35; 95% confidence interval (CI) = 1.28–1.43) and the lowest in winter. Similarly, the rate of admissions for diverticulitis was higher in summer than in winter (OR = 1.23; 95% CI = 1.16–1.31), similar to the findings for acute cholecystitis (OR = 1.23; 95% CI = 1.11–1.36) and acute cholangitis (OR = 1.23; 95% CI = 1.12–1.36).

### 3.3. Seasonal Severity of Each Disease

**Figure 4:**
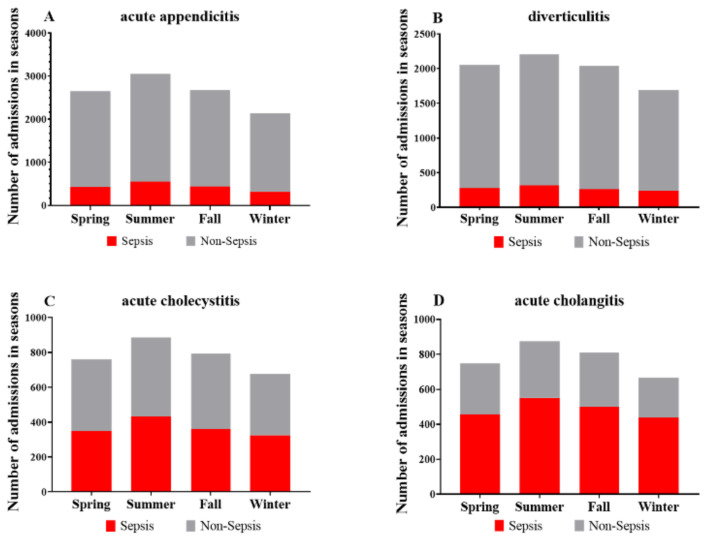
The proportion of cases showing sepsis for each disease across seasons, 2011–2019. (**A**), acute appendicitis, (**B**), diverticulitis, (**C**), acute cholecystitis, (**D**), acute cholangitis.

The proportions of cases showing sepsis as an underlying condition for each disease in all four seasons are shown in Figure 4. The total SOFA score and organ sub-scores for acute appendicitis, diverticulitis, acute cholecystitis, and acute cholangitis showed no meaningful differences across months and seasons. In analyses of variations and regression, no seasonal variation in the incidence of Sepsis-3 as an underlying condition was observed for any of the four diseases.

## 4. Discussion

### 4.1. Principal Findings

Our study showed that the incidence rates of acute appendicitis, diverticulitis, acute cholecystitis, and acute cholangitis in the summer were higher than those in the winter. The seasonal incidence of each disease was consistent with that reported in previous studies [12]. To the best of our knowledge, this is the first study to showed that the incidence rate of acute cholangitis was the highest in summer and lowest in winter. Although the seasonal pattern of infections for the blind-ending tubular structure has been reported, a similar trend of admission rate was observed in acute cholangitis.

### 4.2. Assumed Mechanisms Causing Seasonal Variations of Acute Abdomen

Previous studies have reported that bacterial and viral infections, fiber intake, temperature, humidity, daily sun exposure, and atmospheric pressure were related to seasonal variations in acute appendicitis, with the highest incidence occurring in the summer [18,19,20]. Acute appendicitis is caused by obstruction of the lumen due to lymphoid hyperplasia, fecaliths, and neoplasms. The incidence of infectious gastroenteritis, which can occur in lymphoid hyperplasia, reportedly increases with increasing temperature. In addition, infections by *Escherichia coli*, which was the most common pathogen of acute appendicitis, peaked in the summer [21,22]. These findings could be associated with the higher admission rate during summer. The primary etiological factors for acute cholangitis and cholecystitis are gallstones [23]. Cholestasis and impaired intestinal motility may promote gallstone formation [24]. Dehydration during summer may also influence gallstone formation. Biliary culture tests detected gram-negative bacilli such as *Escherichia coli* and *Klebsiella* spp., which were reported to show a higher incidence in bloodstream infections during summer and a higher incidence with rising temperatures [25,26]. These factors could be associated with the seasonality of acute cholecystitis and cholangitis. Our findings showed the seasonal incidence of acute abdomen in Japan with four distinct seasons and may predict relevant risk factors for diseases in the summer.

### 4.3. Disease Severity across Seasons

A previous study reported that the incidence of sepsis was higher in winter, and the rate of respiratory sepsis was higher in winter. In addition, sepsis due to the gastrointestinal system has been reported to have no seasonality [27]. Our study focused on the severity of each disease; the findings did not show any clear seasonal variations, consistent with the results of previous studies. Studies have suggested that the seasonality of respiratory infection was associated with low temperature, which suppressed host immune responses, but all four diseases in our study were common diseases not related to immune deficiency [28].

### 4.4. Limitations

Our study had several limitations. First, the records of the diagnoses for acute appendicitis, diverticulitis, acute cholecystitis, and acute cholangitis recorded in the MDV database may have included errors because diagnoses recorded in administrative claims databases generally show lower accuracy than those recorded in prospective studies. Similarly, under- or overestimation and misclassification of the underlying conditions may have occurred. Incorrect diagnoses may have occurred in cases where attending physicians recorded ICD-10 diagnoses outside their fields of expertise or image diagnosis was not performed, particularly in mild cases. Second, the acute hospitals included in this study accounted for only 1.2% of the total hospitals in Japan, but the study nevertheless included a larger number of patients than previous studies in Japan. Third, we could not obtain the pathological and diagnostic imaging findings to assess the reliability of the diagnoses, which is likely to lead to an overestimate of cases.

## 5. Conclusions

The findings of this retrospective observational study of 24,708 acute abdomen patients over a nine-year study period in Japan suggested that emergency admissions for acute appendicitis, diverticulitis, acute cholecystitis, and acute cholangitis were highest in the summer. However, no variations were observed in the severity of acute abdomen among the four seasons.

## Figures and Tables

**Table 1:** Patient characteristics.

	All Patients (*n* = 24,708)	Acute Appendicitis (*n* = 10,500)	Diverticulitis (*n* = 7993)	Acute Cholecystitis (*n* = 3114)	Acute Cholangitis (*n* = 3101)
Age, yr	60 (37–77)	37 (19–57)	65 (48–78)	76 (65–84)	79 (70–85)
<18, *n* (%)	2457 (9.9%)	2408 (22.9%)	34 (0.4%)	6 (0.2%)	9 (0.3%)
18–64, *n* (%)	11,206 (45.4%)	6160 (58.7%)	3831 (47.9%)	747 (24.0%)	468 (15.1%)
≥65, *n* (%)	11,045 (44.7%)	1932 (18.4%)	4128 (51.6%)	2361 (75.8%)	2624 (84.6%)
Sex, male, (%)	14,107 (57.1%)	5868 (55.9%)	4698 (58.8%)	1811 (58.2%)	1730 (55.8%)
Charlson comorbidity index	2 (0–5)	1 (0–3)	3 (1–6)	4 (2–8)	6 (3–10)
SOFA score	1 (0–2)	0 (0–1)	0 (0–1)	1 (0–3)	2 (1–3)
Sepsis-3, *n* (%)	6224 (25.2%)	1726 (16.4%)	1090 (13.6%)	1462 (46.9%)	1946 (62.8%)
SOFA score for sepsis-3 patients	3 (2–4)	2 (2–3)	2 (2–3)	3 (2–4)	3 (2–4)
DIC, *n* (%)	732 (3.0%)	154 (1.5%)	196 (2.5%)	173 (5.6%)	209 (6.7%)
ICU admission, *n* (%)	463 (1.9%)	132 (1.3%)	165 (2.1%)	124 (4.0%)	42 (1.4%)
Emergency surgery with general anesthesia *n* (%)	6132 (24.8%)	3989 (38.0%)	675 (8.4%)	911 (29.3%)	557 (18.0%)
Catecholamine use, *n* (%)	744 (3.0%)	226 (2.2%)	103 (1.3%)	123 (3.9%)	292 (9.4%)

Data are expressed as a percentage or median and interquartile range, as indicated. SOFA, Sequential Organ Failure Assessment; CNS, central nervous system; DIC, disseminated intravascular coagulation; ICU; intensive care unit.

**Table 2:** Outcome measures.

	All Patients (*n* = 24,708)	Acute Appendicitis (*n* = 10,500)	Diverticulitis (*n* = 7993)	Acute Cholecystitis (*n* = 3114)	Acute Cholangitis (*n* = 3101)
Mortality, *n* (%)	254 (1.0%)	12 (0.1%)	40 (0.5%)	95 (3.1%)	107 (3.5%)
<18 years, *n* (%)	0/2457 (0%)	0/2408 (0%)	0/34 (0%)	0/6 (0%)	0/9 (0%)
18–64 years, *n* (%)	11/11,206 (0.1%)	0/6160 (0%)	4/3831 (0.1%)	2/747 (0.3%)	5/468 (1.1%)
≥65 years, *n* (%)	243/11,045 (2.2%)	12/1932 (0.6%)	36/4128 (0.9%)	93/2361 (3.9%)	102/2624 (3.9%)
Length of stay, d	7 (4–11)	5 (3–8)	7 (5–10)	12 (7–20)	10 (6–15)

Data are expressed as percent or median and interquartile range, as indicated.

**Table 3:** Analysis of the seasonal admissions for each diagnosis.

	Acute Appendicitis		Diverticulitis		Acute Cholecystitis		Acute Cholangitis	
Season	Odds Ratio (95% CI)	*p* Value	Odds Ratio (95% CI)	*p* Value	Odds Ratio (95% CI)	*p* Value	Odds Ratio (95% CI)	*p* Value
Spring	1.20 (1.14–1.27)	<0.0001	1.18 (1.10–1.26)	<0.0001	1.08 (0.98–1.20)	0.1285	1.08 (0.98–1.20)	0.137
Summer	1.35 (1.28–1.43)	<0.0001	1.23 (1.16–1.31)	<0.0001	1.23 (1.11–1.36)	<0.0001	1.23 (1.12–1.36)	<0.0001
Fall	1.20 (1.13–1.27)	<0.0001	1.16 (1.08–1.23)	<0.0001	1.12 (1.01–1.24)	0.031	1.16 (1.05–1.28)	0.0048
Winter	Reference		Reference		Reference		Reference	

Data are expressed as a percent or mean with 95% confidence interval, as indicated. *p*-Value for trend test using the logistic regression analysis, as appropriate.

## Data Availability

The datasets used and/or analyzed during the current study are available from the corresponding author on reasonable request.

## Supplementary Materials

The following is available online at https://www.mdpi.com/article/10.3390/jpm11121346/s1, Table S1: Modified SOFA score.
