# Supplementary material for: Seasonal Variation and Severity of Acute Abdomen in Japan: A Nine-Year Retrospective Analysis
Source: J Pers Med. 2021 Dec 10;11(12):1346. doi: 10.3390/jpm11121346 (PMC8709094; doi:10.3390/jpm11121346)
Supplement: Supplementary file 1 [file jpm-11-01346-s001.zip › jpm-1496223-supplementary.pdf]

Supplemental Table S1. Modified SOFA score

Modified SOFA score	1	2	3	4
CNS	Japan coma scale = 1, 2, or 3	Japan coma scale = 10 or 20	Japan coma scale = 30 or 100	Japan coma scale = 200 or 300
Liver	Bilirubin > 1.2 mg/dL	Bilirubin > 2.0 mg/dL	Bilirubin > 6.0 mg/dL	Bilirubin > 12.0 mg/dL
Renal	Creatine > 1.2 mg/dL	Creatine > 2.0 mg/dL	Creatine > 3.5 mg/dL	Creatine > 5.0 mg/dL
Coagulation	Platelets < 150×10^3/μl	Platelets < 100×10^3/μL	Platelets < 50×10^3/μL	Platelets < 20×10^3/μL
Cardiovascular		DOA < 5 or any DOB	DOA, 5.1 to 15, AD ≤ 0.1, or NAD ≤ 0.1	DOA > 15, AD > 0.1, or NAD > 0.1
Respiration	Oxygenation	High-flow nasal cannula oxygen or non-invasive positive pressure ventilation	Mechanical ventilation	

SOFA, Sequential Organ Failure Assessment; CNS, central nervous system; DOA, dopamine; DOB, dobutamine; AD, adrenaline; NAD, noradrenaline.
